# Flux tope analysis: studying the coordination of reaction directions in metabolic networks

**DOI:** 10.1093/bioinformatics/bty550

**Published:** 2018-07-02

**Authors:** Matthias P Gerstl, Stefan Müller, Georg Regensburger, Jürgen Zanghellini

**Affiliations:** 1 Department of Biotechnology, University of Natural Resources and Life Sciences, Vienna, Austria, EU; 2 Austrian Centre of Industrial Biotechnology, Vienna, Austria, EU; 3 Faculty of Mathematics, University of Vienna, Vienna, Austria, EU; 4 Institute for Algebra, Johannes Kepler University Linz, Linz, Austria, EU; 5 Austrian Biotech University of Applied Sciences, Tulln, Austria, EU

## Abstract

**Motivation:**

Elementary flux mode (EFM) analysis allows an unbiased description of metabolic networks in terms of minimal pathways (involving a minimal set of reactions). To date, the enumeration of EFMs is impracticable in genome-scale metabolic models. In a complementary approach, we introduce the concept of a flux tope (FT), involving a *maximal* set of reactions (with fixed directions), which allows one to study the coordination of reaction directions in metabolic networks and opens a new way for EFM enumeration.

**Results:**

A FT is a (nontrivial) subset of the flux cone specified by fixing the directions of all reversible reactions. In a consistent metabolic network (without unused reactions), every FT contains a ‘maximal pathway’, carrying flux in all reactions. This decomposition of the flux cone into FTs allows the enumeration of EFMs (of individual FTs) without increasing the problem dimension by reaction splitting. To develop a mathematical framework for FT analysis, we build on the concepts of sign vectors and hyperplane arrangements. Thereby, we observe that FT analysis can be applied also to flux optimization problems involving additional (inhomogeneous) linear constraints. For the enumeration of FTs, we adapt the *reverse search* algorithm and provide an efficient implementation. We demonstrate that (biomass-optimal) FTs can be enumerated in genome-scale metabolic models of *B.cuenoti* and *E.coli*, and we use FTs to enumerate EFMs in models of *M.genitalium* and *B.cuenoti*.

**Availability and implementation:**

The source code is freely available at https://github.com/mpgerstl/FTA.

**Supplementary information:**

Supplementary data are available at *Bioinformatics* online.

## 1 Introduction

The development of constraint-based modeling (CBM) approaches contributed tremendously to our understanding of metabolic processes, in particular, to the analysis of genome-scale metabolic models (GSMMs). Combined with CBM approaches, GSMMs provide a mechanistic basis for our understanding of the genotype-phenotype relationship.

For the analysis of GSMMs, two branches within the CBM spectrum turned out to be most successful: flux-balance analysis and elementary flux mode (EFM) analysis. Both method families use stoichiometric information and consider the linear equalities and inequalities for the reaction rates (fluxes) that arise from the steady-state assumption and irreversibility constraints. Whereas flux-balance analysis identifies *optimal* solutions (under additional linear constraints) and remains computationally practicable even at genome scale, EFM analysis describes all *feasible* solutions (the flux cone) in terms of minimal metabolic pathways. Due to the combinatorial nature of EFM enumeration, such an analysis faces severe computational challenges already for medium-scale metabolic models (Jungreuthmayer *et al.*, 2013). Despite major advances in algorithm design (Gagneur and Klamt, 2004; Hunt *et al.*, 2014; Terzer and Stelling, 2008; van Klinken and Willems van Dijk, 2016), EFM enumeration for GSMMs is not practicable to date. Hence other approaches focused on the enumeration of subsets of EFMs characterized by particular qualities (De Figueiredo *et al.*, 2009; Kaleta *et al.*, 2009).

In metabolic networks with reversible reactions, (thermodynamically feasible) EFMs can be grouped into largest (thermodynamically) consistent sets (LTCSs) (Gerstl *et al.*, 2016). For all EFMs within one LTCS, the directions of all reactions are fixed (as determined by the Gibbs free energy). Importantly, every flux mode can be written as a sum of EFMs from one LTCS. In fact, a fundamental result of EFM analysis states that every flux mode can be written as a *conformal* sum of EFMs, that is, if a component of the flux mode has a certain sign, then this component has the same sign (or is zero) in all EFMs involved (Müller and Regensburger, 2016; Urbanczik and Wagner, 2005). In our previous work, it remained open whether LTCSs can be defined without referring to EFMs and computed without enumerating all EFMs beforehand. In the present paper, we show that this is indeed possible.

We introduce the novel concept of a *flux tope* (FT) as a (nontrivial) subset of the flux cone specified by fixing the directions of all reversible reactions. Obviously, every flux mode is contained in a FT, that is, the flux cone is decomposed into FTs. A feasible combination of reaction directions naturally corresponds to a *sign vector* (having –, 0, or + entries) of the flux cone, and every FT corresponds to a support-maximal sign vector of the flux cone. In fact, the term ‘tope’ comes from the theory of oriented matroids, where it refers to a maximal sign vector of a linear subspace (Bachem and Kern, 1992; Bokowski, 2006). Whereas an EFM represents a minimal pathway (involving a minimal set of reactions), a FT contains a maximal ‘pathway’ (involving a maximal set of reactions). As EFMs, FTs need not be thermodynamically feasible, and we discuss the definition and computation of thermodynamically feasible FTs (corresponding to LTCSs) in the outlook. Ultimately, FT analysis can be used to study the coordination of reaction directions in GSMMs, that is, the thermodynamic repertoire of cellular metabolism.

Most importantly, the enumeration of FTs (as opposed to EFMs) is computationally practicable even at larger scale. Our implementation is based on the *reverse search* algorithm for cell enumeration in *hyperplane arrangements* (Avis and Fukuda, 1996; Fukuda, 2016). Moreover, FTs can be used to enumerate EFMs in GSMMs with reversible reactions. Indeed, FTs can be computed first, and EFMs (of individual FTs) can be enumerated efficiently (without increasing the problem dimension by reaction splitting) in a second step.

## 2 Materials and methods

### 2.1 Sign vectors

For a vector $x\in R^{n}$, we define the *sign vector*$\text{sign}(x)\in {\left\{-,0,+\right\}}^{n}$ by applying the sign function component-wise, that is,

(1) $$\text{sign}{\left(x\right)}_{i}=\text{sign}(x_{i})\;\;\text{for}\;\;i=1,\ldots ,n,$$

and we write

(2) sign(S)={sign(x)|x∈S}

for a subset $S\subseteq R^{n}$.

The relations 0 < – and 0 < + induce a partial order on {–, 0,+}^*n*^: for sign vectors $\xi ,\eta \in {\left\{-,0,+\right\}}^{n}$, we write ξ≤η if the inequality holds component-wise and say that ξ*conforms* to η. Analogously, for $x\in R^{n}$ and $\xi \in {\left\{-,0,+\right\}}^{n}$, we say that ***x*** conforms to ξ if sign(x)≤ξ. E.g.

$$\text{sign}\;\left(\begin{array}{r} -1\\ 0\\ 2 \end{array}\right)=\left(\begin{array}{c} -\\ 0\\ + \end{array}\right)\leq \left(\begin{array}{c} -\\ -\\ + \end{array}\right)=\text{sign}\;\left(\begin{array}{r} -2\\ -1\\ 1 \end{array}\right),$$

that is, (–, 0, +)^*T*^ conforms to (–,–,+)^*T*^, and (–1, 0, 2)^*T*^ conforms to (–, 0, +)^*T*^ (trivially) and (–,–,+)^*T*^. Given a subset $S\subseteq R^{n}$ and a sign vector $\xi \in {\left\{-,0,+\right\}}^{n}$, we define

(3) $$S_{\leq \xi }=\{x\in S|\text{sign}(x)\leq \xi \},$$

the subset of *S* conforming to ξ. (In the application to metabolic networks below, the set *S* is the flux cone, and the sign vector ξ is a maximal sign vector of the flux cone, fixing the directions of all reactions.)

Finally, we call the vectors $x,y\in R^{n}$*conformal* if there exists a sign vector $\xi \in {\left\{-,0,+\right\}}^{n}$ such that sign(x),sign(y)≤ξ or, equivalently, if *x_i_ y_i_* ≥ 0 for i=1,…,n.

### 2.2 Metabolic networks

A *metabolic network* is given by *m* internal metabolites, *r* reactions and the corresponding stoichiometric matrix $N\in R^{m\times r}$, which contains the net stoichiometric coefficients of each metabolite in each reaction. The sets of irreversible and reversible reactions are given by $I_{\text{irr}}\subseteq \{1,\ldots ,r\}$ and $I_{\text{rev}}=\{1,\ldots ,r\}\setminus I_{\text{irr}}$, respectively. A vector of reaction rates that satisfies the steady-state and irreversibility constraints is called a *flux mode*. In geometric terms, a flux mode is an element of the *flux cone*

(4) $$C=\{v\in R^{r}|\mathrm{Nv}=0\;\text{and}\;v_{i}\geq 0\;\text{for}\;i\in I_{\text{irr}}\},$$

a polyhedral cone defined by the nullspace of the stoichiometric matrix and nonnegativity conditions.

### 2.3 Flux topes

An EFM e∈C is a support-minimal nonzero flux mode, and every element of the ray {λe|λ>0} is an EFM, too. With respect to the partial order on {–, 0,+}^*r*^ defined above, the sign vector sign(e) is a minimal nonzero element of

(5) sign(C)={sign(v)|v∈C},

the set of all sign vectors of the flux cone. Conversely, a minimal nonzero sign vector σ∈sign(C) determines the ray

$$\begin{array}{l} C_{\leq \sigma }=\{v\in C|\text{sign}(v)\leq \sigma \}\\ =\{v\in C|\text{sign}(v)=\sigma \}\\ =\{\lambda e|\lambda >0\}, \end{array}$$

where e∈C is some EFM with sign(e)=σ. Analogously, a maximal sign vector τ∈sign(C) determines the pointed subcone

(6) $$C_{\leq \tau }=\{v\in C|\text{sign}(v)\leq \tau \},$$

which we call a *flux tope (FT)*.

A FT $C_{\leq \tau }$ consists of all flux modes that conform to the defining sign vector τ∈sign(C), in particular, it contains all conforming EFMs. Indeed, EFMs are extreme rays of FTs, and this property may serve as a definition of EFMs (Klamt *et al.*, 2017; Müller and Regensburger, 2016).

### 2.4 Consistency

A flux cone is called *consistent* (Acuña *et al.*, 2009) if every reaction (in every possible direction) is supported by a flux mode, that is, if for every i∈{1,…,r} there exists v∈C such that *v_i_* > 0 and, additionally, for every *i* ∈ *I*_rev_ there exists v′∈C such that $v\prime_{i}<0$. We say that a flux mode has *full support*, if all its components are nonzero.

Proposition 1. If a flux cone is consistent, then every reaction (in every possible direction) is supported by a flux mode with full support.

Proof. Let *C* be a consistent flux cone and i∈{1,…,r}. Then there exists v∈C such that *v_i_* > 0. Suppose ***v*** does not have full support, that is, *v_j_* = 0 for some *j *≠* i*. By consistency, there exists w∈C such that *w_j_* > 0. Now, consider the convex combination u=(1−λ)v+λw∈C. For sufficiently small 0 < *λ* <  1, sign(u)>sign(v), in particular, *u_i_*, *u_j_* > 0. Repetition of the argument eventually yields a flux mode with full support.

Finally, let *i* ∈ *I*_rev._ Then there exists v∈C such that *v_i_* < 0, and a flux mode with full support can be constructed as above. □

We say that a FT $C_{\leq \tau }$ has full support, if the defining maximal sign vector τ∈sign(C) has full support, that is, if $\tau \in {\left\{-,+\right\}}^{r}$.

Proposition 2. If a flux cone is consistent, then all FTs have full support.

Proof. Let *C* be a consistent flux cone. Suppose there exists a FT $\;C_{\leq \tau }$ with a maximal sign vector τ∈sign(C) that does not have full support, and let $v\in C_{\leq \tau }$ with sign(v)=τ. By consistency, there exists w∈C with full support. Now, consider the convex combination u=(1−λ)v+λw∈C. For sufficiently small 0 < *λ* <  1, ***u*** has full support and sign(u)>sign(v)=τ, contradicting that τ is maximal. □ 

Note that a flux cone can be made consistent using flux variability analysis, see Section 3.1.

### 2.5 Hyperplane arrangements

Let the columns of the matrix $K\in R^{r\times d}$ form a basis of the nullspace of the stoichiometric matrix ***N***, and hence NK=0. Further, let $K^{i}\in R^{d}$ for i=1,…,r denote the *i*th row of ***K*** and

(7) $$h^{i}=\{x\in R^{d}|K^{i}x=0\}\;\;\text{for}\;\;i=1,\ldots ,r$$

be the corresponding *(central) hyperplane*. Then, every flux mode v∈C can be written as

(8) v=Kx,

where $x\in R^{d}$ is unique and $v_{i}=K^{i}x\geq 0$ for *i* ∈ *I*_irr._ Most importantly, $\text{sign}(v)\in {\left\{-,0,+\right\}}^{r}$ describes the positions of ***x*** with respect to the hyperplanes $h^{1},\ldots ,h^{r}$. In particular, a sign vector of the flux cone with full support (defining a FT) corresponds to a *cell* of the *hyperplane arrangement* that satisfies the irreversibility constraints.

For a general central hyperplane arrangement of *r* hyperplanes in $R^{d}$, there is a well-known upper bound for the number of cells: Out of 2^*r*^ full sign vectors, $2\sum_{i=0}^{d-1}\left(\begin{array}{c} r-1\\ i \end{array}\right)$ correspond to cells (Buck, 1943; Fukuda, 2016). This upper bound simplifies to 2^*r*^ if *d *≥* r*. In case of irreversibility constraints, where $r=|I_{\text{rev}}|+|I_{\text{irr}}|$, we have the obvious upper bound $2^{\left|I_{\text{rev}}\right|}$ for the number of FTs. In case $|I_{\text{rev}}|=0$, there is only one FT.

### 2.6 A toy model

**Fig. 1.**
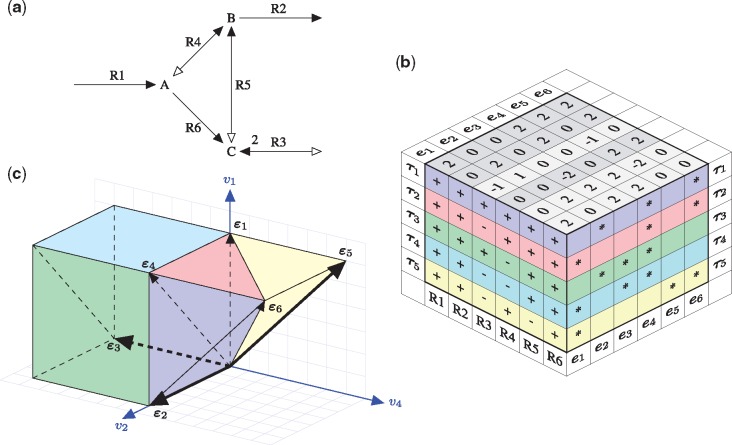
(**a**) Toy model with three internal metabolites (A, B, C) and six reactions, where $|I_{\text{irr}}|=3$ reactions are irreversible (R1, R2, R6) and $|I_{\text{rev}}|=3$ are reversible (R3, R4, R5). Forward and backward directions are indicated by full and empty arrow heads, respectively. Reaction R3 produces two molecules of C (stated next to the arrow head), all other stoichiometric coefficients are one. (**b**) Three-dimensional table listing the EFMs $e_{i}$ and the FTs $\tau_{j}$. Containment of EFMs in FTs is marked by ‘*’. Note that out of $2^{\left|I_{\text{rev}}\right|}=8$ full sign vectors, only five define FTs, while the remaining three do not correspond to flux modes, see also Figure 2. (**c**) EFMs and FTs projected on the flux components *v*_1_, *v*_2_, *v*_4_ with colors as in table (b). The projected EFMs ${ɛ}_{i}$ are depicted by (full and dashed) arrows, and their components are highlighted in the top plane of table (b) and listed in Equation (12). The projected EFMs generate the projected FTs and the projected hyperplane (separating the FTs). In particular, the projected EFMs ${ɛ}_{2},\;{ɛ}_{3}$ and ${ɛ}_{5}$ (thick arrows) generate the projected flux cone (Color version of this figure is available at *Bioinformatics* online.)

We consider the small network displayed in Figure 1a. It consists of three internal metabolites and six reactions. The resulting stoichiometric matrix amounts to

(9) $$N=\left(\begin{array}{rrrrrr} 1 & 0 & 0 & -1 & 0 & -1\\ 0 & -1 & 0 & 1 & 1 & 0\\ 0 & 0 & 2 & 0 & -1 & 1 \end{array}\right).$$

A basis of the nullspace of ***N*** is given by the columns of the matrix

(10) $$K=\left(\begin{array}{rrr} 1 & 0 & 0\\ 0 & 1 & 0\\ -\frac{1}{2} & \frac{1}{2} & 0\\ 0 & 0 & 1\\ 0 & 1 & -1\\ 1 & 0 & -1 \end{array}\right).$$

Every flux mode can be written as v=Kx with a unique $x\in R^{3}$. Since the submatrix of ***K*** consisting of the rows 1, 2 and 4 (corresponding to the reactions R1, R2 and R4) is the identity matrix, we get

(11) $$v=K\left(\begin{array}{l} v_{1}\\ v_{2}\\ v_{4} \end{array}\right).$$

Now, the irreversible reactions R1, R2 and R6 define the nonnegativity conditions $v_{1}\geq 0,\;v_{2}\geq 0$, and *v*_1_ – *v*_4_ ≥ 0 and shape the flux cone, whereas the reversible reactions R3, R4 and R5 determine the hyperplanes $-\frac{1}{2}v_{1}+\frac{1}{2}v_{2}=0,\;v_{4}=0$, and *v*_2_ – *v*_4_ = 0 and divide the flux cone into FTs. The resulting five FTs are listed in Figure 1b. The projection of the FTs on the flux components *v*_1_, *v*_2_ and *v*_4_ is depicted in Figure 1c.

The six (generating) EFMs $e_{i}$ of the toy network are listed in Figure 1b, and their projections ${ɛ}_{i}$ are depicted in Figure 1c. According to Equation (11), we can write them as

(12) $$(e_{1},\ldots ,e_{6})=K({ɛ}_{1},\ldots ,{ɛ}_{6})=K\left(\begin{array}{rrrrrr} 2 & 0 & 0 & 2 & 2 & 2\\ 0 & 2 & 0 & 2 & 0 & 2\\ 0 & 0 & -2 & 0 & 2 & 2 \end{array}\right).$$

Each FT is generated by three EFMs. (This is the smallest possible number since the dimension of the nullspace is three.) The EFMs $e_{4},\;e_{1}$ and $e_{6}$ are contained in the largest number of FTs (four and three, respectively), see Figure 1b and c. They generate the most ‘central’ FT $\tau_{2}$ (depicted in pink), having the largest number of neighbours (three). The EFMs $e_{2}$ and $e_{3}$ are contained in two FTs each. Together with the above EFMs, they generate four (out of five) FTs. The remaining EFM $e_{5}$ is contained only in the most ‘peripheral’ FT $\tau_{5}$, having only one adjacent FT. As opposed to the other FTs, flux vectors in $\tau_{5}$ use reaction R5 in reverse direction.

### 2.7 Reverse search

If (i) the flux cone is consistent, then all maximal sign vectors have full support, by Proposition 2. If (ii) the nullspace matrix does not contain rows which are multiples of each other, then hyperplanes are distinct, and cells can be enumerated using *reverse search* (Avis and Fukuda, 1996). The algorithm starts from a cell in the hyperplane arrangement (represented by a full sign vector) and recursively checks all *adjacent* full sign vectors (differing in exactly one component) whether they represent cells.

In our implementation, we use the idea that only adjacent full sign vectors need to be checked, however, for efficiency reasons, we adapt the algorithm. In particular, we do not operate on the hyperplane arrangement, but directly on full sign vectors, see Section 3.2.

In the following, we assume (i) and (ii) which can be ensured using appropriate pre-processing, see Section 3.1.

### 2.8 Flux optimization

In flux-balance analysis, one often optimizes linear combinations of reaction rates under box constraints, i.e. one solves linear programs (LPs)

(13) $$\max\;c^{T}v\;s.t.\;v\in P,$$

defined on the *flux polyhedron*

(14) $$P=\{v\in R^{r}|\mathrm{Nv}=0\;\text{and}\;\ell_{i}\leq v_{i}\leq u_{i}\;\text{for}\;i=1,\ldots ,r\},$$

where $\ell_{i},u_{i}\in [-\infty ,+\infty ]$. The lower and upper bounds define a corresponding flux cone *C*, in particular, *i* ∈ *I*_irr_ if and only if *ℓ_i_* ≥ 0. If *ℓ_i_* = –*∞* or 0 and *u_i_* = +*∞* for all i∈{1,…,r}, then *P *=* C*, otherwise P⊂C.

Let $v^{*}$ be an optimal flux and $d=c^{T}v^{*}$ the corresponding optimal value. Then $P^{*}=\{v\in P|c^{T}v=d\}$ is the polyhedron of optimal fluxes. As for the flux cone *C*, FTs and consistency can be defined for the optimal flux polyhedron $P^{*}$ (Klamt *et al.*, 2017). After ensuring consistency using flux variability analysis, all FTs of the flux polyhedron have full support and correspond to cells in a (non-central) hyperplane arrangement that satisfy the box constraints. Finally, after ensuring that hyperplanes are distinct (see Section 3.1), FTs can be enumerated using reverse search.

**Fig. 2.**
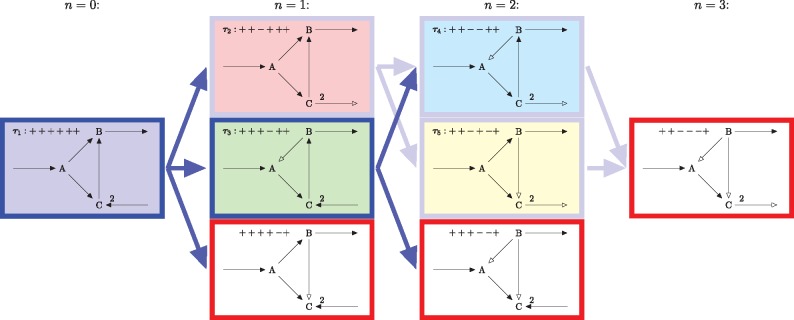
Enumeration of FTs for the toy network in Figure 1 (with colors as in Figure 1). Out of $2^{\left|I_{\text{rev}}\right|}=8$ full sign vectors, only five define FTs. Two FTs maximize the flux through reaction R2 (dark blue frames), three are sub-optimal (light blue frames). Three full sign vectors do not represent a FT (red frames), since either C is only produced or B is only consumed. Sign vectors are depicted as nodes of a directed acyclic graph (arranged in levels *n *=* *0 through *n *=* *3) with directed edges pointing from ’parent’ to ’child’ sign vectors (Color version of this figure is available at *Bioinformatics* online.)

In our toy model (Fig. 1), assume upper bounds for the uptake reactions R1 and R3 in Figure 1a, in particular, *v*_1_ ≤ 10 and *v*_3_ ≤ 10. Then the projected flux cone in Figure 1c becomes a polyhedron with $v_{2}\leq 30,\;v_{3}\geq -5,\;v_{4}\leq 10$ and $v_{5}\geq -10$. Still, since EFM $e_{3}$ (the internal cycle) is not constrained by the uptake reactions, there is no lower bound for *v*_4_ (and no upper bounds for *v*_5_ and *v*_6_). As a consequence, FTs $\tau_{1},\;\tau_{2}$ and $\tau_{5}$ become bounded, whereas $\tau_{3}$ and $\tau_{4}$ remain unbounded (for negative *v*_4_). When the flux through the product reaction R2 is optimized, then the maximum *v*_2_ = 30 is attained at flux distributions in FTs $\tau_{1}$ and $\tau_{3}$, see again Figures 1c and 2. Note that optimal solutions are contained in adjacent FTs, in particular, $\tau_{1}$ and $\tau_{3}$ are separated by the hyperplane *v*_4_ = 0, and the direction of reaction R4 is not determined by the optimum.

### 2.9 Genome-scale metabolic models

We study GSMMs of *Mycoplasma genitalium*, *i*PS189+ (Suthers *et al.*, 2009 including recent modifications by Hartleb *et al.*, 2016), *Blattabacterium cuenoti* Bge, *i*CG238 (González-Domenech *et al.*, 2012) and *Escherichia coli* K-12 MG1655, *i*JR904 (Reed *et al.*, 2003). For *i*PS189+ and *i*CG238, we allow the consumption of all nutrients for which uptake reactions are present in the model. For *i*JR904, we model growth on minimal medium [ammonium, hydrogen(+), oxygen, phosphate, sulfate] with glucose as the sole carbon source. A summary of the algebraic characteristics of the models is given in Table 1. All models are available at https://github.com/mpgerstl/FTA.

**Table 1.** Algebraic characteristics of consistent GSMMs: dimensions *m *×* r* of the stoichiometric matrix ***N***, dimension of the nullspace with basis ***K***, d=rank(K), and number of independent reactions, *r*_ind_. (Numbers in brackets refer to the numbers of reversible reactions.)

Organism	model ID	*m *×* r*	*d*	EFMs	run time	*r*_ind_	FTs	run time	*r*_ind_	FTs (max.BM)	run time
*M.genitalium*	*i*PS189+	271 × 277 (21)	28	3 252 686	10.3 h	83 (13)	672	1.0 s	83 (7)	48	<1.0 s
*B.cuenoti*	*i*CG238	306 × 350 (45)	51	c.i.	—	137 (31)	60 226 956	29.8 h	137 (10)	270	<1.0 s
*E.coli*	*i*JR904	450 × 667 (53)	233	c.i.	—	432 (49)	c.i.	—	432 (27)	11 796 480	34.8 h

*Note*: Computational results: number of EFMs [computed by FluxModeCalculator (van Klinken and Willems van Dijk, 2016)], number of FTs (computed by our implementation), and number of FTs that maximize biomass production (max.BM).

c.i., computationally infeasible.

## 3 Implementation

### 3.1 Pre-processing

We use flux variability analysis (Mahadevan and Schilling, 2003) to make the flux cone consistent. That is, we remove all reactions that cannot carry nonzero steady-state flux and change all reversible reactions into irreversible that cannot carry flux in both directions.

Further, we identify an initial FT determined by a maximal sign vector of the flux cone. By consistency, this sign vector has full support and, after changing the directions of reversible reactions having a minus entry, it has only plus entries.

Finally, we determine reaction dependencies. We compute a basis matrix for the nullspace of the stoichiometric matrix, using the nullspace method of the R package pracma, and determine rows (dependent reactions) that are multiples of other rows (independent reactions).

### 3.2 Efficient enumeration of flux topes

To check if a full sign vector $\tau \in {\left\{-,+\right\}}^{r}$ (with *τ_i_* = + for *i* ∈ *I*_irr)_ determines a FT, we check the feasibility of the LP

(15) $$\mathrm{Nv}=0,\;\ell \leq \tau_{i}v_{i}\leq u\;\;\text{for}\;\;i=1,\ldots ,r.$$

For numerical reasons, we set lower and upper bounds, *ℓ* = 10^–^^6^ and *u *=* *10^3^, respectively, and a tolerance of the LP solver of at most 10^–^^10^.

The algorithm starts with the sign vector having only plus entries. In the first step, it visits all full sign vectors having one minus entry in an independent reversible reaction (and all reactions depending on it) and checks their feasibility, using the above LP (see Fig. 2). In the second step, the algorithm visits all feasible, full sign vectors having two minus entries in an independent reversible reaction, and so on.

More specifically, in step *n*, the algorithm starts with the set of all feasible full sign vectors having *n* – 1 minus entries (the ‘parent’ sign vectors), and visits all full sign vectors with *n* minus entries (the ‘child’ sign vectors). Note that ‘child’ sign vectors can be reached from several ‘parent’ sign vectors. If a sign vector is visited for the first time, its feasibility is checked using the above LP and stored in a tree of bit patterns (one bit, plus or minus, for each independent reversible reaction), in order to avoid the repetition of the feasibility check. The algorithm terminates if there are no feasible full sign vectors having *n* minus entries or if *n* reaches the number of independent reversible reactions. For an illustration of our implementation, see Figure 2 and Supplementary Table S1.

Our enumeration algorithm can be threaded efficiently. In particular, checking the feasibility of ‘child’ sign vectors for a given ‘parent’ sign vector forms an independent task.

We implemented the algorithm in C. LPs are solved with CPLEX. The source code is available at https://github.com/mpgerstl/FTA. Unless otherwise stated, computations were carried out using six threads on a Xeon^®^ E5-1650v3 CPU with DDR4 RAM modules running on Debian 8.

## 4 Results

### 4.1 FTs correspond to maximal sets of conformal EFMs

We analyzed a GSMM of *M.genitalium*, *i*PS189+ (Hartleb *et al.*, 2016; Suthers *et al.*, 2009) and enumerated all FTs and all EFMs. (The enumeration of all EFMs was possible since the model is sufficiently small.) More than 3 million EFMs were found, which are contained in only 672 FTs, see Table 1. The FTs were enumerated within 1 s, whereas EFM computation took 10 h.

We verified that the 672 FTs correspond to maximal sets of conformal EFMs (having matching signs). Thereby, we first computed the set of all EFMs and formed the maximal sets of conformal EFMs using a mixed integer LP described in Gerstl *et al.* (2016), which was previously used for the computation of LTCS from the set of EFMs. We also computed the sets of EFMs for all individual FTs and found that their union equals the set of all EFMs.

We conclude that in network containing reversible reactions (i) FTs can be enumerated efficiently, (ii) few FTs condense the information contained in many EFMs and (iii) EFMs can be computed using FTs.

### 4.2 FT analysis may be feasible when EFM analysis is not

We studied a GSMM of *B.cuenoti*, a mutualistic, bacterial endosymbiont living in fat cells of cockroaches. The model *i*CG238 (González-Domenech *et al.*, 2012) is significantly larger than *i*PS189+, and a full EFM analysis is infeasible with current methods. However, we were able to enumerate all FTs within 30 h and found 60.2 × 10^6^ FTs, see Table 1.

**Fig. 3.**
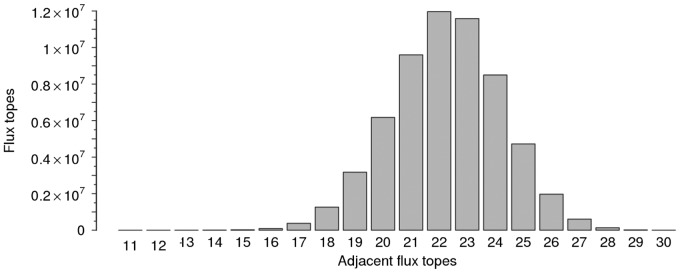
Frequency of the number of adjacent FTs, computed in *i*CG238

We note that the number of FTs is much smaller than the obvious upper bound 2^31^ = 2.15 × 10^9^, where 31 is the number of independent reversible reactions. To attain this upper bound, each FT would need to have 31 adjacent FTs. However, most frequently, a FT has only 22 adjacent FTs, see Figure 3.

### 4.3 Optimal FTs can be enumerated in GSMMs

For the model *i*CG238 (González-Domenech *et al.*, 2012), we were further interested in fluxes that maximize biomass production. As described in Section 2.8, we enumerated the FTs of the optimal flux polyhedron. We found that, out of the 60 million FTs of the flux cone, only 270 are FTs of the optimal flux polyhedron, see Table 1. In fact, the optimal FTs could be identified within 1 s, without first enumerating all FTs (taking 30 h) and then selecting the optimal ones. We verified that both approaches result in the same set of biomass-optimal FTs.

The decrease in the number of FTs from 60 million to 270 is a consequence of additional irreversibility constraints arising from the optimality condition. While the model *i*CG238 contains 31 independent reversible reactions, biomass-optimality enforces 21 additional irreversibility constraints leaving only ten reactions reversible, see Table 1. Interestingly, out of all amino acid transport reactions, only the exchange of Alanine remained reversible. All other amino acids cannot be produced when *B.cuenoti* is growing optimally.

**Fig. 4.**
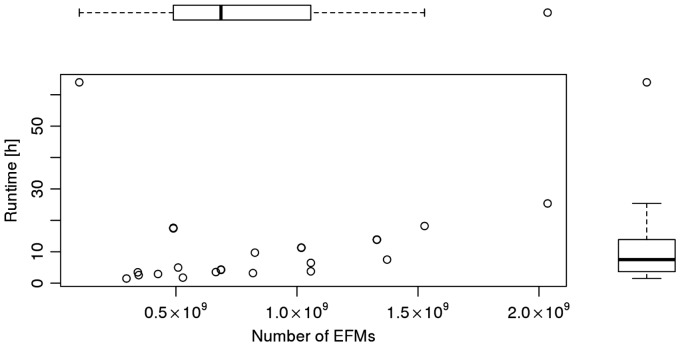
Runtime (of EFM enumeration) versus number of EFMs for 27 randomly selected, biomass-optimal FTs, computed in *i*CG238

To complete the study of the model *i*CG238, we randomly selected 10% of the biomass-optimal FTs and performed an EFM analysis. All FTs contained around 10^9^ EFMs, see Figure 4; however, the run times for EFM enumeration varied strongly, ranging from 1 h to more than 60 h in one extreme case.

Finally, we analyzed a GSMM of *E.coli*, *i*JR904 (Reed *et al.*, 2003). We enumerated all biomass-optimal FTs and found around twelve million FTs within less than 35 h runtime. Interestingly, the number of FTs computed in each step of our algorithm is distributed normally, see left panels in Figure 6 and Supplementary Figure S2. Indeed, the same distribution was found for *B.cuenoti*, *i*CG238, see Supplementary Figure S3 in the supplement.

**Fig. 5.**
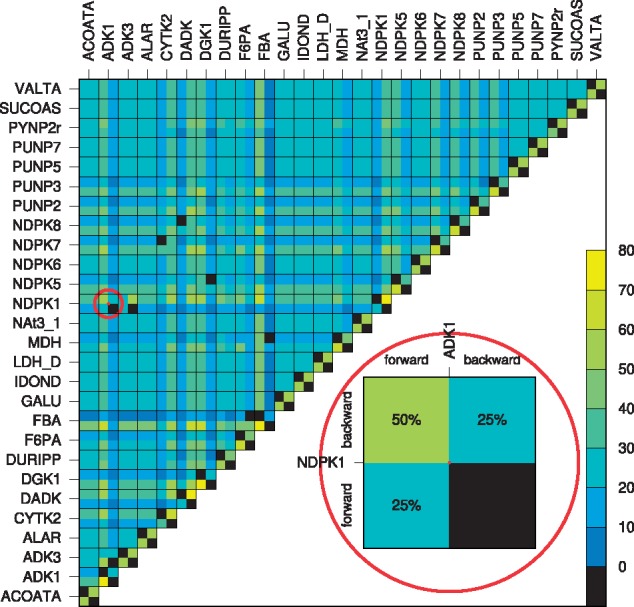
Relative frequency of pairs of reaction directions in biomass-optimal FTs of *i*JR904. (Tick labels correspond to reaction identifiers in *i*JR904.) Every cell corresponds to a pair of reversible reactions and is divided in four squares corresponding to the possible combinations of reaction directions. E.g. 50% of all biomass-optimal FTs are supported by reaction NDPK1 in backward and reaction ADK1 in forward direction (see inset). Black squares depict unfeasible pairs of reaction directions (Color version of this figure is available at *Bioinformatics* online.)

Next, we studied the frequency of reaction directions in biomass-optimal FTs of *i*JR904. The direction of fructose-bisphosphate aldolase (FBA) turned out to be most rigid, with the forward direction being used in 80% of the FTs. On the other hand, 12 (out of the 27) reversible reactions were most flexible, showing no preference for forward or backward directions, see the diagonal in Figure 5. In fact, Figure 5 illustrates the coordination of reaction directions for *pairs* of reversible reactions. Only seven (out of $\left(\begin{array}{c} 2\times 27\\ 2 \end{array}\right)=1431$) pairs of reaction directions are infeasible (black squares in the off-diagonal cells in Fig. 5), thereby highlighting the plasticity of metabolic networks. While most infeasible pairs occurred within the nucleotide salvage pathway, some also occurred across different pathways, e.g. the infeasible pair of malate dehydrogenase (MDH) and fructose-bisphosphate aldolase (FBA) from the tricarboxylic acid cycle and glycolysis, respectively.

**Fig. 6.**
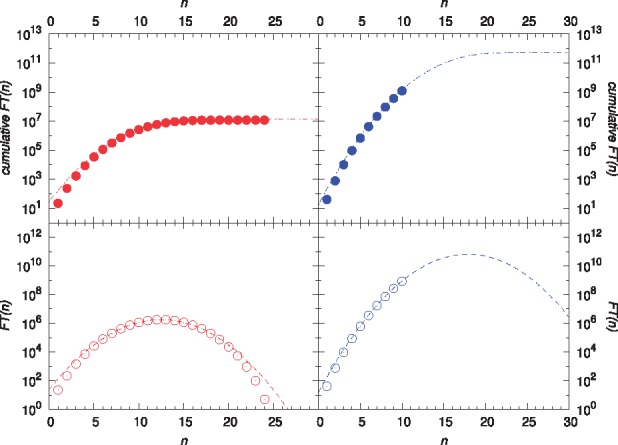
Cumulative and incremental number of FTs as a function of the step size *n* (top and bottom panels, respectively). In particular, number of biomass-optimal and all FTs (left and right panels, respectively), computed in *i*JR904 (*E.coli*). Dashed lines represent fits to normal distributions. Parameter values are listed in Supplementary Table S2

The enumeration of *all* FTs turned out to be computationally infeasible. In fact, the enumeration of all FTs up to step *n *=* *11 (see Fig. 6) required two months and 260 GB memory, thereby using 20 threads on two Intel^®^ Xeon^®^ E5-2650v3 CPUs with DDR4 RAM modules running on CentOS 7. Assuming that the incremental number of FTs is distributed normally, we estimated the total number of FTs to be around 10^12^, see top-right panel in Figure 6. This prediction is by two orders of magnitude lower than the upper bound determined by the number of independent reversible reactions. The quality of the fit was evaluated for *i*CG238 (*B.cuenoti*) and biomass-optimal FTs of *i*JR904 (*E.coli*), where already after a few steps the predictions are within a 50% range of the true value, cf. Supplementary Figure S4.

## 5 Discussion

In this work, we introduced the novel concept of a flux tope (FT). For a consistent metabolic network, a FT is a full-dimensional pointed subcone of the flux cone, specified by fixing the directions of all (reversible) reactions. In particular, every FT contains a full ‘pathway’, carrying flux in all reactions. Whereas flux variability analysis allows to study the feasible directions of individual reactions, FT analysis allows to study all feasible (or all optimal) combinations of reaction directions. We developed a mathematical framework for FT analysis, building on the concepts of sign vectors and hyperplane arrangements, we provided an efficient algorithm for the enumeration of FTs, we demonstrated that FTs can be enumerated in large metabolic networks, and we used FTs to enumerate EFMs in metabolic networks with reversible reactions. Ultimately, we are interested in FTs that are both stoichiometrically and thermodynamically feasible and hence characterize the thermodynamic repertoire of cellular metabolism.

To efficiently enumerate FTs, we build on the correspondence between FTs and cells in a (central) hyperplane arrangement. In particular, we adapt the *reverse search* algorithm for cell enumeration in hyperplane arrangements. Reverse search is both *compact* and *output-polynomial*. (Recall that an algorithm is compact if its space requirement is polynomial in the input size only and output-polynomial if its runtime is polynomial in both input and output size.) Moreover, it constantly produces output (not just upon completion). As it turns out, enumerating cells in the hyperplane arrangement (7) is problematic. In particular, solving LPs involving the (dense) null-space matrix ***K*** is slow. Hence, we directly solve the LPs (15) involving the (sparse) stoichiometric matrix ***N***. Further, we trade some space requirements for smaller runtime and store the solutions of LPs to avoid repeated computations. Finally, we change the algorithm from depth-first to breadth-first search. This allows to investigate neighborhoods of a given FT, if the enumeration of *all* FTs is computationally infeasible or if the reversion of reaction directions increases an objective function (e.g. biomass). In fact, it was suggested that reversing reaction directions can improve strain performance (Nishikawa *et al.*, 2008). Moreover, coordination of reaction directions is key to the study of emergent properties in cross-feeding communities. Currently, it is unclear if members of a community adjust their metabolism in an optimal manner, and unbiased methods like FT analysis are required to identify essential interactions between species (Gottstein *et al.*, 2016).

For EFM enumeration, a metabolic network is often ‘reconfigured’ by splitting reversible reactions, and one considers the resulting higher-dimensional network involving irreversible forward and backward reactions. This approach is not practicable for FT enumeration. For the reconfigured system, there is exactly one (trivial) FT. To identify the FTs of the original system, additional constraints have to be added: For every reversible reaction, either the forward or the backward flux has to be zero. Due to the enforced zero fluxes, the FT enumeration problem is not an LP (but a mixed integer LP), and (efficient) reverse search cannot be used.

All models under study have significantly fewer FTs than EFMs. In fact, in the GSMM of *B.cuenoti*, every single FT has more EFMs than the whole network has FTs. This is in contrast to general hyperplane arrangements, in which there are least as many topes (sign vectors with maximal support) as vertices (sign vectors with minimal support) (Fukuda *et al.*, 1991). We conjecture that the lower number of FTs compared to EFMs is a typical feature of GSMMs; a detailed comparison will be the scope of further work. Currently, metabolic pathway analysis is restricted to medium-scale models since the number of EFMs explodes with the size of a model. FTs helps to accommodate this problem in two ways: (i) there are fewer FTs than EFMs, and (ii) they can be enumerated more efficiently. (Recall that the complexity of the double description method for EFM enumeration is not even known).

Finally, the enumeration of FTs opens up a new way for enumerating EFMs in GSMMs. The flux cone is the union of all FTs, which can be subject to EFM analysis, individually. For a given FT, the directions of all (reversible) reactions are fixed, and the double description method can be used without increasing the problem dimension by reaction splitting. On our machines, a conventional EFM analysis of *i*CG238 (*B.cuenoti*) was infeasible due to memory restrictions. Still, we were able to enumerate all EFMs of individual FTs, cf. Figure 4, which suggests the parallel enumeration of EFMs for all FTs. Clearly, a naive parallelization is inefficient, since EFMs are typically contained in several FTs. Especially EFMs contained in FTs with many adjacent cells are shared frequently. Tests with *i*PS189+ indicate that, on average, an EFM is enumerated more than 100 times. Yet, despite the frequent repetitions, the total CPU run time (compared to a standard EFM analysis) increased only by a factor of ten. Further work is needed to make a FT-based EFM enumeration competitive in terms of run time.

## 6 Outlook: thermodynamically feasible FTs

Recently, it has been shown that many EFMs are thermodynamically infeasible and hence irrelevant for the characterization of metabolic phenotypes (Gerstl *et al.*, 2016, 2015a,b; Jungreuthmayer *et al.*, 2015; Peres *et al.*, 2017). The same constraints apply to FTs. In our toy model, the FTs $\tau_{3}$ and $\tau_{4}$ contain the thermodynamically infeasible EFM $e_{3}$ (the internal cycle), cf. Figures 1b and 2, and hence they are irrelevant biologically. A single thermodynamically infeasible EFM leads to the elimination of two FTs, that is, thermodynamic constraints reduce the number of FTs even more than the number of EFMs.

A thermodynamically feasible FT represents one possible combination of reaction directions and contains all corresponding pathways. Thereby, the thermodynamic feasibility of a FT is determined by the metabolite concentrations via the Gibbs free energy. By cellular control of the metabolite concentrations, a FT can be reached and the corresponding pathways can be activated.

A first generalization of our enumeration algorithm involves the elimination of FTs that do not contain any thermodynamically feasible flux mode: either by straightforward post-processing or by further adaptation of *reverse search*. In the end, we are not just interested in FTs (defined by *full* sign vectors) that contain thermodynamically feasible flux modes (possibly with smaller sign vectors), but rather in *thermodynamically feasible* FTs (defined by *maximal* sign vectors). The latter definition leads to combinatorial problems which require further theoretical analysis and algorithmic developments.

## Funding

MPG and JZ were supported by the Austrian BMWD, BMVIT, SFG, Standortagentur Tirol, Government of Lower Austria, and Business Agency Vienna through the Austrian FFG-COMET-Funding Program, project 23071. SM and GR were supported by the Austrian Science Fund, project P28406 and P27229, respectively.

*Conflict of Interest*: none declared.

## Supplementary Material

Supplementary Figures
