# Supplementary material for: Flux tope analysis: studying the coordination of reaction directions in metabolic networks
Source: Bioinformatics. 2018 Jul 2;35(2):266–73. doi: 10.1093/bioinformatics/bty550 (PMC6330010; doi:10.1093/bioinformatics/bty550)
Supplement: Supplementary Figures [file bty550_supplementary_figures.pdf]

S1 Supplementary material

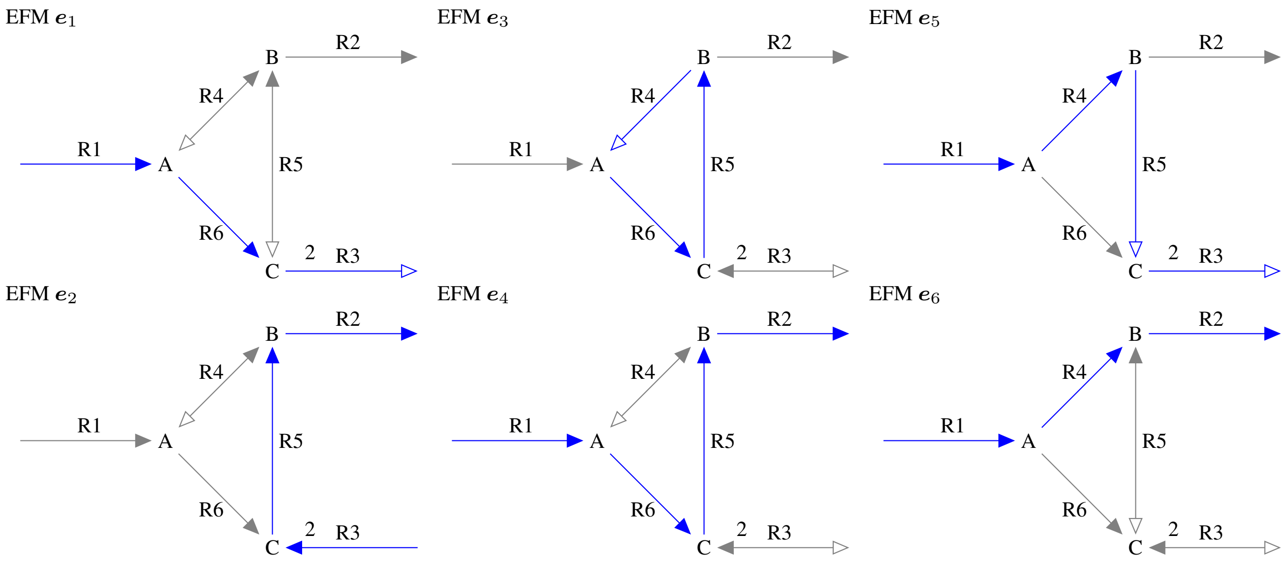

Fig. **S1**: Set of EFMs for the toy model in Figure 1. Reactions in support of an EFM are drawn in blue. EFMs are numbered in accordance to (12).

Table **S1**. Performance indicators of the algorithm described in section 3.2 applied to the toy model described in section 2.6 for enumerating all FTs and all FTs that maximize the flux through reaction R2. The number of adjacency tests (ATs) corresponds to the number of directed edges (dark blue edges in case of max.R2) in Figure 2. The number of LPs corresponds to the number of sign vectors visited.

	ATs	LPs	FTs
FTs	9	7	5
FTs (max.R2)	5	5	2

Data fitting

We used the (scaled) normal distribution

$$f(n) = \frac{\alpha}{\sqrt{2\pi\sigma^2}} \exp\left[-\frac{(n-\mu)^2}{2\sigma^2}\right],$$

with the parameters α, μ, and σ to fit the incremental number of FTs as a function of the step size n. Fitting was performed with the function `NonlinearModelFit` in Mathematica 11, Version 11.0.1.0 on a Mac OS X x86.

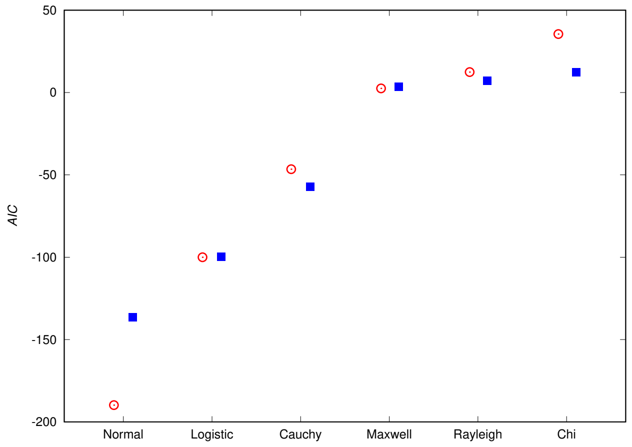

Fig. **S2**: Relative fit quality for different fit functions of the *E. coli* FT distribution in the lower panels of Figure 6 (cycle, biomass-optimal FTs; square, all FTs) as measured by the Akaike information criterion (AIC). AIC values were evaluated with the function `NonlinearModelFit` in Mathematica 11. Note that the parameters for the chi-distribution were constraint.Thus AIC values may not reliable when calculated with `NonlinearModelFit`.

Table **S2**. Predicted values for the fitting parameters used in Figure 6, Figure **S2** and Figure **S3**. Abbreviations: AIC, Akaike information criterion; max.BM, biomass-optimal; MEDIAN, median; SD, standard deviation;

	B. cuenoti		*E. coli*	
	FTs (max.BM)	FTs	FTs (max.BM)	FTs
MEDIAN(α)	274	6.04×10^7	1.18×10^7	4.40×10^{11}
SD(α)	0.650	3.72×10^4	0	0
MEDIAN(μ)	4.53	14.1	12.5	17.9
SD(μ)	0.340	0.749	0	0
MEDIAN(σ)	1.71	2.84	2.63	2.69
SD(σ)	0.155	0.188	0	0
MEDIAN(AIC)	-43.2	-230	-190	-136
SD(AIC)	9.18	25.9	0	0
Sample size	270	30	1	1

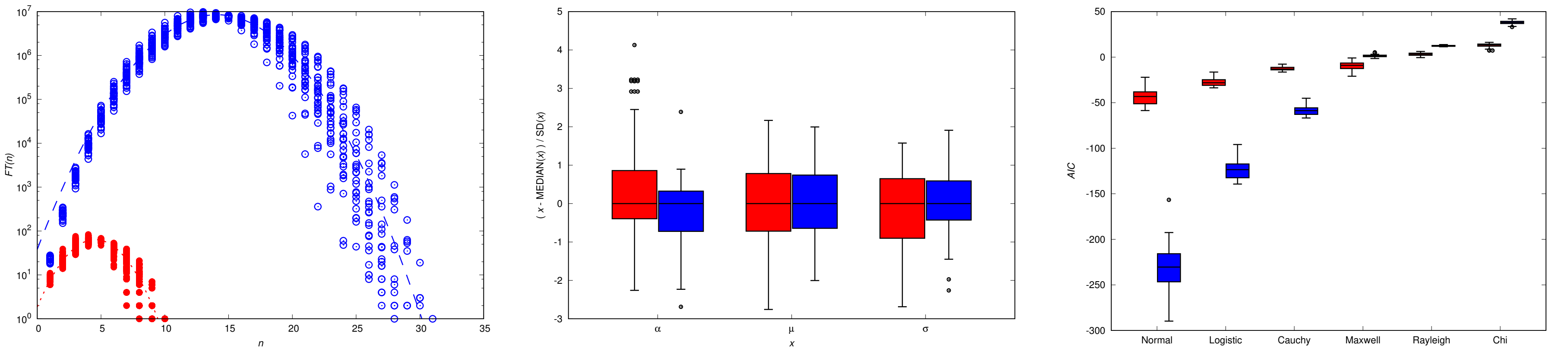

(a) FT density function as function of the step size n for all possible starting FTs (open circles). The dashed line represents a fit of the data using a scaled normal distribution, see section **S1**. The parameter values are given by their median value as stated in Figure **S3**b.

(b) Normalized fitting parameters α, μ, and σ of the scaled normal distribution used to fit the individual runs displayed in Figure **S3**a. Abbreviations: MEDIAN, median; SD, standard deviation;

(c) Akaike information criterion (AIC) evaluated for different fit functions of the data in Figure **S3**a. AIC values were obtained with the function `NonlinearModelFit` in Mathematica 11, Version 11.0.1.0 on a Mac OS X x86 (32-bit, 64-bit Kernel).

Fig. **S3**: FT density functions and various fitting parameters in *B. cuenoti* for biomass-optimal FTs (red) and all FTs (blue). The variations are caused by starting the enumeration from different initial FTs.

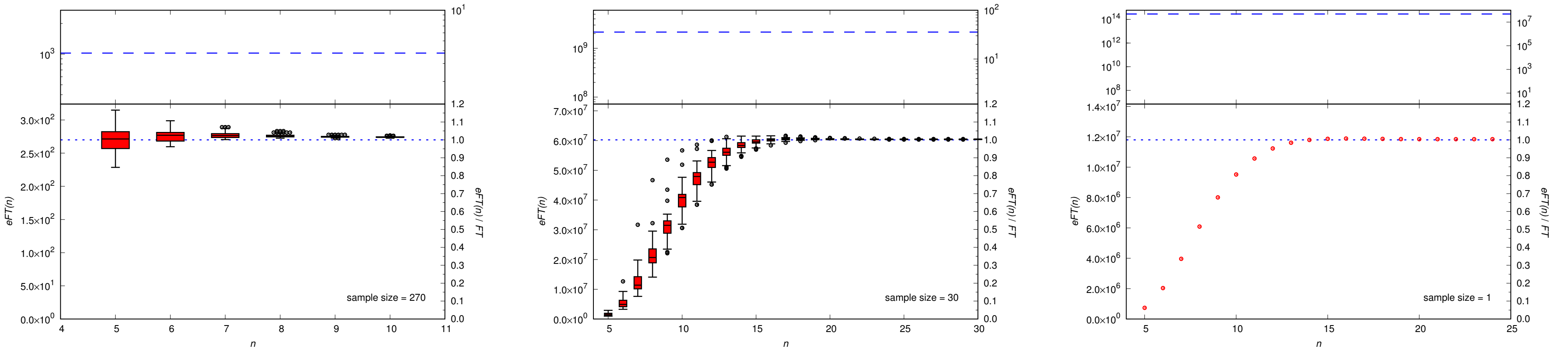

(a) biomass-optimal eFTs in *B. cuenoti*, *i*CG238.

(b) eFTs in *B. cuenoti*, *i*CG238.

(c) biomass-optimal eFTs in *E. coli*, *i*JR904.

Fig. **S4**: Variation in the estimated number of FTs (eFT) as function of the step size n in various organisms. Data are plotted in true values and normalized to the actual number of FTs in the models (left and right y-axis, respectively). Variations were estimated based on randomly selecting feasible starting FTs and enumerating all FTs from there. Sampling sizes are listed at the bottom of each plot. For $eFT(n)$ all data points up to step n were used in the fitting procedure. The dotted and dashed lines indicate the position of the actual number of FTs and the (naive) upper bound 2^r, respectively, with r being the number of independent reversible reactions as listed in Table 1. Note that the upper third of each plot is scaled logarithmically, while the lower two-third of each plot is scaled linearly.

"fluxtopes" — 2018/5/30 — 15:02 — page S-3 — #11
